# The Efficacy and Safety of Mirabegron for the Treatment of Neurogenic Lower Urinary Tract Dysfunction: A Systematic Review and Meta-analysis

**DOI:** 10.3389/fphar.2021.756582

**Published:** 2021-11-18

**Authors:** Dongxu Zhang, Fengze Sun, Huibao Yao, Xingjun Bao, Di Wang, Yuanshan Cui, Jitao Wu

**Affiliations:** ^1^ Department of Urology, Yantai Yuhuangding Hospital, Qingdao University, Yantai, China; ^2^ Department of Urology, Beijing Tiantan Hospital, Capital Medical University, Beijing, China

**Keywords:** meta-analysis, mirabegron, neurogenic lower urinary tract dysfunction, RCT, randomized controlled trial, systematic review

## Abstract

**Background and Objective:** Over the past few years, mirabegron has been increasingly used as a therapeutic option for neurogenic lower urinary tract dysfunction. Here, we carried out a meta-analysis to investigate the efficacy and safety of mirabegron for the treatment of neurogenic lower urinary tract dysfunction.

**Methods:** We used a range of databases to retrieve randomized controlled trials (RCTs) relating to mirabegron in patients with neurogenic lower urinary tract dysfunction: PubMed, Embase, and Cochrane Library; our strategy conformed to the PICOS (populations, interventions, comparators, outcomes, and study designs) strategy.

**Results:** Our analyses involved four RCTs involving 245 patients. We found that mirabegron treatment resulted in a significant improvement in bladder compliance [mean difference (MD) = 19.53, 95% confidence interval (CI): 14.19 to 24.87, P < 0.00001], urinary incontinence episodes (MD = −0.78, 95% CI: −0.89 to −0.67, *P* < 0.00001) and Incontinence Quality of Life (I-QOL) (MD = 8.02, 95% CI: 3.20 to 12.84, *P* = 0.001). Significant differences were detected in terms of Patient Perception of Bladder Condition (PPBC) (MD = −0.54, 95% CI: −1.46 to 0.39, *P* = 0.26) and urinary urgency episodes (MD = −0.72, 95% CI: −3.1 to 1.66, *P* = 0.55). With regard to safety, there were no significant differences between mirabegron and control groups in terms of the incidence of drug-related adverse events [odds ratio (OR): 0.83, 95% CI: 0.43 to 1.59, *P* = 0.57], arrhythmias (OR: 1.27, 95% CI: 0.37 to 4.38, *P* = 0.70), hypertension (OR: 0.70, 95% CI: 0.13 to 3.82, *P* = 0.68), or post-voiding residual volume (MD: 1.62, 95% CI: −9.00 to 12.24, *P* = 0.77).

**Conclusion:** Mirabegron is an efficacious and safe treatment for patients with neurogenic lower urinary tract dysfunction.

## Introduction

Patients suffering from spinal cord injury (SCI) and neurological disorders (e.g., multiple sclerosis (MS) and Parkinson’s disease) often present with neurogenic lower urinary tract dysfunction (NLUTD) (Stöhrer et al., 2009; Harris and Lemack, 2016). The typical clinical symptoms of NLUTD usually manifest as dysuria, urgency, urinary incontinence, and impaired bladder emptying. Patients with severe NLUTD can develop renal failure and complicated urinary tract infections and may even die. At present, anticholinergic (antimuscarinic) drugs are recommended as the first-line treatment for NLUTD. Although some studies have reported that anticholinergic (antimuscarinic) medications can effectively improve urodynamic parameters in patients with NLUTD (Madhuvrata et al., 2012; Sugiyama et al., 2017), these medicines are associated with side effects (e.g., dry mouth and constipation) that limit their use in the long term (Averbeck and Madersbacher, 2011; Manack et al., 2011; Wagg et al., 2012). Therefore, there is a clear need to develop novel, effective, and safe therapeutic modalities for NLUTD.

Mirabegron, a β3-adrenoceptor agonist, is commonly applied to treat idiopathic overactive bladder in the clinic and works by stimulating β3-adrenergic receptors to induce detrusor relaxation (Kashyap and Tyagi, 2013). Compared with anticholinergic (antimuscarinic) drugs, mirabegron has similar levels of efficacy but with superior safety (Maman et al., 2014; Chapple et al., 2017). More recently, mirabegron has been gradually applied for the treatment of NLUTD. However, few evidence-based studies have been conducted on the feasibility of using mirabegron as a treatment for NLUTD. In view of their superior safety profile, mirabegron is expected to become a new option for the treatment of NLUTD.

In this systematic review and meta-analysis, we assessed the efficacy and safety of mirabegron for the treatment of NLUTD to provide a feasible reference for clinical medication. Our study adhered to the Preferred Reporting Items for Systematic Reviews and Meta-Analyses checklist.

## Methods

### Search Strategy

Three of the authors identified randomized controlled trials (RCTs) relating to the impact of mirabegron in the treatment of NLUTD from the PubMed, Embase, and Cochrane Library databases, in accordance with the PICOS (populations, interventions, comparators, outcomes, and study designs) strategy; the search strategy is summarized in Table 1. Our database searches included the following search terms: NLUTD, SCI, neurological disorders (MS and Parkinson’s disease), mirabegron, and RCTs. Our analysis was registered with PROSPERO (Reference: CRD42021256235). References from the included articles were also reviewed by the three authors to identify additional relevant articles.

**TABLE 1:** Search strategy according to populations, interventions, comparators, outcomes, and study designs (PICOS).

	Population	Intervention	Comparator	Outcomes	Study design
Inclusion criteria	Patients with neurogenic lower urinary tract dysfunction	Mirabegron	Placebo	Patient Perception of Bladder Condition (PPBC)	Randomized controlled trials
				Cystometric capacity	
				24-h pad weight test	
				Bladder compliance, volume at first neurogenic detrusor overactivity	
				Complications, systolic pressure, diastolic pressure, heart rate	
Exclusion Criteria	Patients with non-neurogenic lower urinary tract dysfunction. Anticholinergics in the treatment of the neurogenic lower urinary tract dysfunction in patients. Individuals with indwelling catheters/epicystostomy. Patients with urologic surgery within the past year	Not performed	Not performed	Dairy number of urinations	Letters, comments, reviews, qualitative studies
				Dairy fluid intake	
				MusiQoL score	
				Overactive bladder symptom score	
				Treatment satisfaction questionnaires (TSQ)	

### Inclusion Criteria

To be included in our study, the RCTs needed to satisfy the following criteria: 1) the study analyzed the effect of mirabegron on NLUTD, 2) full-text content was available, and 3) the study provided complete and precise data (including the sample size of participants and the results of each indicator). There were stricter inclusion and exclusion criteria for RCTs, compared with other prospective and retrospective studies.

### Quality Assessment

The quality of the selected RCTs was assessed by applying the Jadad scale (Alejandro, 1998). In addition, the assessment method included patient allocation, the concealment of allocation, blinding methodology, and the number of patients who were lost to follow-up. In accordance with the guidelines published in the Cochrane Handbook for Systematic Reviews of Interventions V.5.1.0 (DerSimonian and Laird, 1986), we classified the quality of each study as follows: 1) the study achieved all quality criteria with a low-risk of bias, 2) the study achieved most quality criteria with a moderate risk of bias, and 3) the study achieved few quality criteria with a high risk of bias. All authors achieved good levels of agreement when applying this classification.

### Data Extraction

We extracted a range of valuable information from each of the RCTs: 1) the name of the first author; 2) the study type; 3) the sample size of each group; 4) the treatment modality; 5) the dosage and time of treatment; and 6) the study outcome, including bladder compliance, Incontinence-Quality of Life (I-QOL), urinary incontinence episodes, urinary urgency episodes, Patient Perception of Bladder Condition (PPBC), the incidence of drug-related adverse events, arrhythmias, hypertension, and post-voiding residual volume.

### Statistical and Meta-Analysis

We performed statistical analysis using Review Manager software (RevMan, version 5.3.0, Cochrane Collaboration) (Higgins and Green, 2008). Differences in bladder compliance; the mean score for the I-QOL and PPBC; and the incidence of drug-related adverse events, arrhythmias, hypertension, and post-voiding residual volume were used to investigate the efficacy of mirabegron for the treatment of NLUTD. Continuous data were evaluated by mean difference (MD) and dichotomous data are expressed by odds ratios (ORs) with 95% confidence intervals (CIs) (DerSimonian and Laird, 2015). When the *p* value was greater than 0.05, the study was regarded as being homogenous. A fixed-effects model was applied to homogenous studies. In contrast, a random-effects model was applicable to heterogeneous studies. We used the I^2^ statistic to test for inconsistency. A *p* value <0.05 was considered to indicate statistical significance.

## Results

### Characteristics of Eligible Studies

**FIGURE 1:**
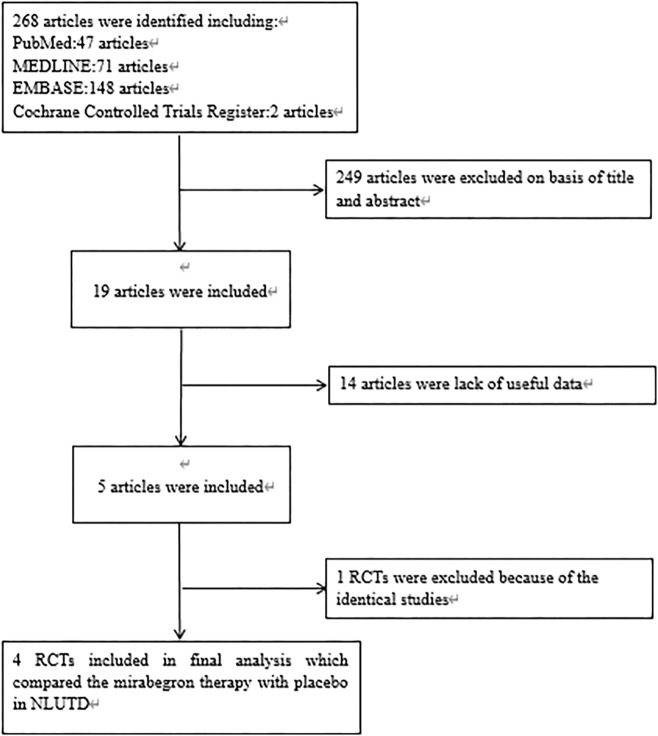
Flowchart of the study selection process. RCT, randomized controlled trials; NLUTD, neurogenic lower urinary tract dysfunction.

After applying the inclusion/exclusion criteria, a total of 286 articles were identified from the databases. First, we screened the titles and abstracts; this led to the removal of 249 articles. When considering the remaining 19 articles, we excluded 14 articles because useful data were missing. One article was eliminated due to duplication. Finally, our analyses involved four high-quality RCTs (Zachariou et al., 2017; Krhut et al., 2018; Welk et al., 2018; Cho et al., 2021). Figure 1 shows a flowchart that presents the selection process. Study features and patient characteristics are given in Table 2.

**TABLE 2:** Study and patient characteristics.

Study	Country	Design	Therapy in experimental group	Therapy in control group	Simple size		Method	Time of therapy (weeks)	Dosage (mg)	Inclusion population
					Trial	Control				
Krhut et al. (2018)	Czech	RCT	Mirabegron	Placebo	32	34	Oral	6	50	Individuals 18–65 years old with NDO arising from SCI or MS; individuals who had experienced SCI at least 12 months before entering the study; subjects with MS who were neurologically stable throughout the preceding 6 months; and subjects who were willing and able to have their concomitant anticholinergic medications withdrawn
Welk et al. (2018)	Canada	RCT	Mirabegron	Placebo	16	16	Oral	10	50	Individuals >18 years old with either a non-acute SCI or MS, and bothersome urinary symptoms (frequency, urgency, and urgency incontinence) and at least one episode of urgency/unaware incontinence during the 3-day voiding diary
Cho et al. (2021)	South Korea	RCT	Mirabegron	Placebo	58	59	Oral	12	50	Eligible patients >20 years old who were diagnosed with Parkinsonism by neurologists; OAB symptoms for 4 weeks or more, OAB symptom score (OABSS) questionnaire total scores ≥3 or higher with a response to Question 3 on urgency of ≥2, and a score of ≤7 on the activities of daily living measured on the expanded disability status scale, which meant that the patients were not necessarily restricted to a bed or wheelchair
Zachariou et al. (2017)	Greece	RCT	Mirabegron + desmopressin	Desmopressin	15	15	Oral	12	50	Between November 2015 and January 2017, 60 patients (20 men and 40 women ≥18 years old) with confirmed MS diagnosis and symptoms of NDO were eligible for screening and study enrolment

RCT, randomized controlled trials; NDO, neurogenic detrusor overactivity; SCI, spinal cord injury; MS, multiple sclerosis; OAB, overactive bladder.

### The Quality of Eligible Studies

The included studies were all RCTs; three of these were randomized, double-blind, and placebo-controlled trials (Krhut et al., 2018; Welk et al., 2018; Cho et al., 2021). The quality grade of three of the included RCTs (Krhut et al., 2018; Welk et al., 2018; Cho et al., 2021) was rated as A; one RCT (Zachariou et al., 2017) was rated as B. One study failed to complete follow-up (Cho et al., 2021), and four patients were lost to follow up. Further details relating to study quality are given in Table 3.

**TABLE 3:** Quality assessment of individual study.

Study	Allocation sequence generation	Allocation concealment	Blinding	Loss to follow-up	Calculation of sample size	Statistical analysis	Level of quality	ITT analysis
Krhut et al. (2018)	A	A	A	0	Yes	ANCOVA	A	No
Blayne Welk et al. (2018)	A	A	A	0	Yes	ANCOVA	A	Yes
Cho et al. (2021)	A	A	A	4	Yes	ANCOVA	A	Yes
Zachariou et al. (2017)	A	A	B	0	Yes	ANCOVA	B	No

A, all quality criteria met (adequate): low risk of bias; B, most quality criteria met (adequate): moderate risk of bias; ITT, intention-to-treat; ANCOVA, analysis of covariance.

### Efficacy

#### Patient Perception of Bladder Condition

**FIGURE 2:**
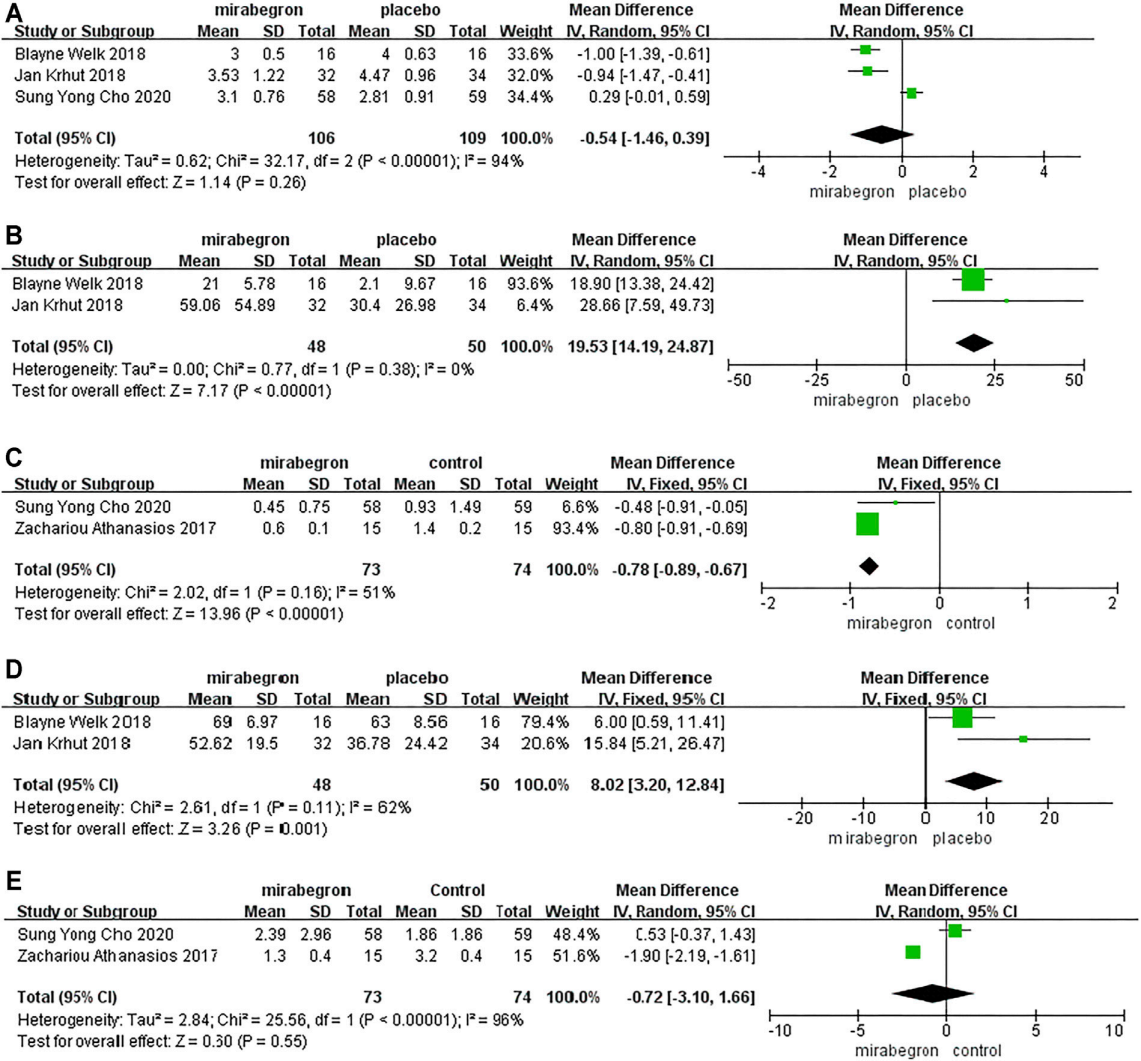
Forest plots showing changes in **(A)** patient perception of bladder condition (PPBC), **(B)** bladder compliance **(C)** urinary incontinence episodes, **(D)** Incontinence Quality of Life (I-QOL), and **(E)** urinary urgency episodes.

Three RCTs analyzed the differences in PPBC across the 352 patients (the mirabegron group consisted of 106 patients, whereas the placebo group consisted of 109 patients) (Figure 2A). Because of *p* < 0.05, we performed a random-effects model; this showed a MD of –0.54 (95% CI: 1.46 to 0.39, I^2^ = 94%, Chi-squared value = 32.17, *p* = 0.26). Our analysis indicated that the effect of mirabegron on PPBC was similar to that of the placebo.

### Bladder Compliance

Two RCTs reported differences in the bladder compliance of 98 patients (48 in the mirabegron group and 50 in the placebo group) (Figure 2B). A random-effects model showed that patients experienced significantly improved bladder compliance following treatment with mirabegron (MD = 19.53; 95% CI: 14.19 to 24.87, *p* ≤ 0.00001).

### Urinary Incontinence Episodes

Two RCTs reported differences in the urinary incontinence episodes of 147 patients (73 in the mirabegron group and 73 in the control group) (Figure 2C). A fixed-effects model indicated that mirabegron significantly improved urinary incontinence episodes in patients with NLUTD (MD = −0.78, 95% CI: −0.89 to −0.67, *p* < 0.00001).

### Incontinence Quality of Life

Two RCTs reported differences in the bladder compliance of 98 patients (48 in the mirabegron group and 50 in the placebo group). Pooled results from a fixed-effects model showed that a statistically significant improvement was recorded in the mirabegron group in terms of the I-QOL scores (MD = 8.02, 95% CI: 3.20 to 12.84, *p* = 0.001) (Figure 2D).

### Urinary Urgency Episodes

Two RCTs reported differences in the urinary urgency episodes of 147 patients (73 in the mirabegron group and 74 in the control group). Pooled results from a random-effects model suggested that the mirabegron group did not differ significantly from that of the control group with regard to improving urinary urgency episodes (MD = −0.72, 95% CI: −3.1 to 1.66, *p* = 0.55) (Figure 2E).

### Safety

#### Adverse Events

**FIGURE 3:**
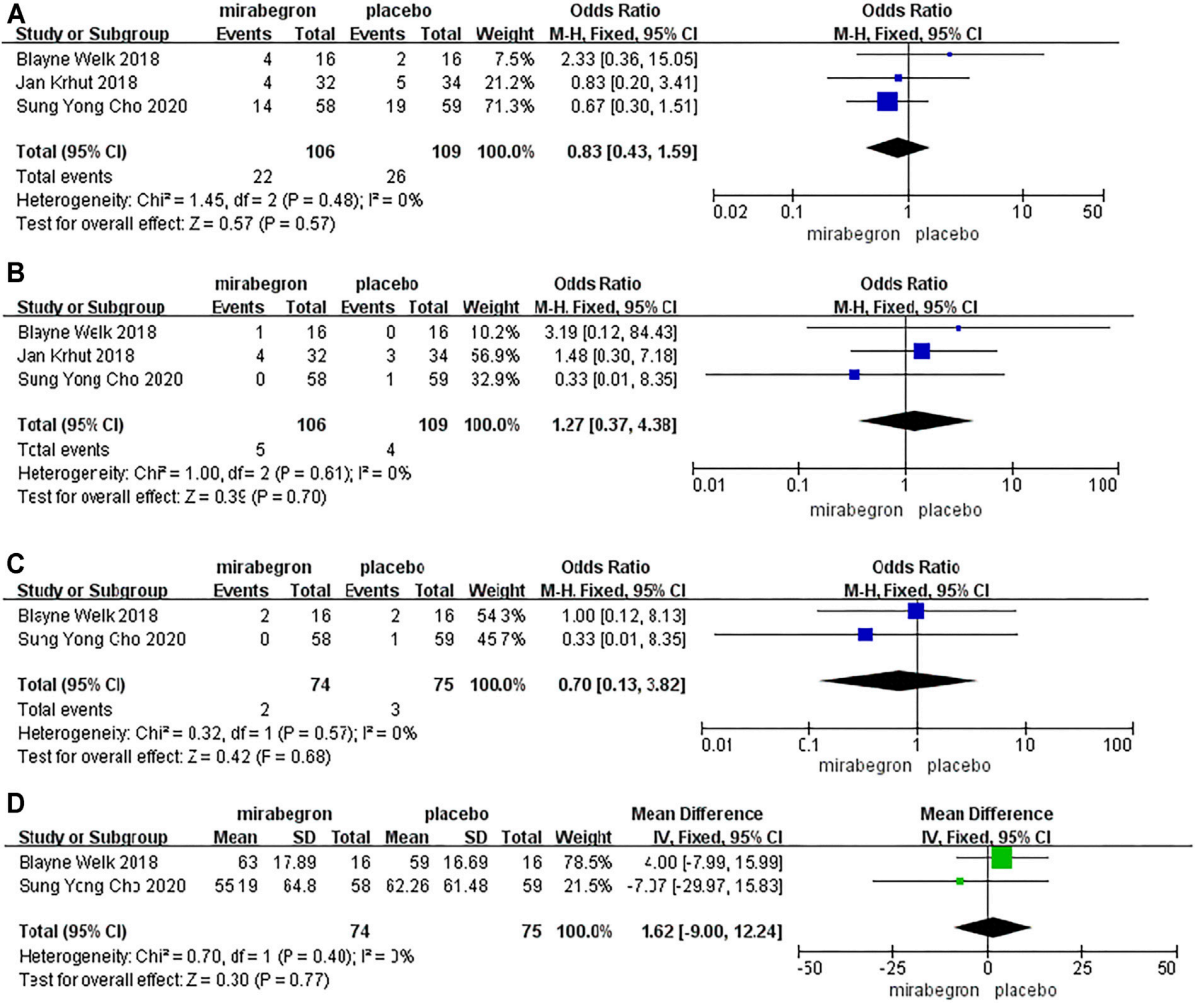
Forest plots showing changes in **(A)** adverse events, **(B)** heart rate, **(C)** blood pressure, and **(D)** post-voiding residual volume.

Because of *p* > 0.05, we performed a fixed-effects model to compare the occurrence of drug-related adverse events between the two groups from three RCTs (Figure 3A). The model indicated that the OR was 0.83, the 95% CI was 0.43 to 1.59, the I^2^ was 0%, and the Chi-squared value was 1.45 (*p* = 0.57), thus indicating that there was no significant difference between the two groups with regard to the occurrence of drug-related adverse events.

#### Heart Rate

Because of *p* > 0.05, we performed a fixed-effects model to analyze the incidence of abnormal heart rate between the two groups from three RCTs (106 patients received mirabegron, whereas 109 patients received placebo treatment) (Figure 3B). The model indicated that the OR was 1.27, the 95% CI was 0.37 to 4.38, the I^2^ was 0%, and the Chi-squared value was 1.00 (*p* = 0.70), thus indicating that the mirabegron and placebo groups were similar in terms of the incidence of abnormal heart rate.

#### Blood Pressure

Two RCTs, including 149 patients (74 in the mirabegron group and 75 in the placebo group), evaluated the risk of abnormal blood pressure (Figure 3C). We utilized a fixed-effects model to analyze these data as *p* > 0.05. The model indicated that the OR was 0.70, the 95% CI was 0.13 to 3.82, the I^2^ was 0%, and the Chi-squared value was 0.32 (*p* = 0.68), thus indicating that there were no significant differences between the two groups with regard to abnormal blood pressure.

#### Post-Voiding Residual Volume

Two RCTs, including 149 patients (74 received mirabegron treatment and 75 received placebo treatment), analyzed post-voiding residual volume (Figure 3D). We used a fixed-effects model to analyze these data, as *p* > 0.05. There was no significant difference between the two groups with regard to post-voiding residual volume (MD = −1.62; 95% CI: −9.00 to 12.24, *p* = 0.77).

## Discussion

Previous epidemiological surveys have shown that the prevalence of SCI in Europe was 0.298%, whereas that of MS was 0.11% (Kingwell et al., 2013; Lee et al., 2014). Developing countries have also been shown to be associated with a high prevalence (2.189%) of cerebrovascular accidents (Przydacz et al., 2017). Studies have also shown that 57%–83% of stroke patients will develop lower urinary tract symptoms just 1 month after cerebrovascular accident (Besiroglu et al., 2015). NLUTD arises from any alteration of the normal neural control mechanisms and can be the consequence of a number of nervous system diseases: SCI, MS, and Parkinson’s. The lower urinary tract is made up of the bladder and the urethra and implements its biological function *via* the storage and voiding of urine. Any neurological lesions or injuries that occur in this complex pathway may contribute to NLUTD. Of the numerous complications of NLUTD, renal failure is the leading cause of mortality (Groen et al., 2016). In addition, lower urinary tract symptoms can exert a serious negative impact on the quality of life. Until now, the management of NLUTD has remained as a major challenge facing the field of urology.

For many years, anticholinergic (antimuscarinic) drugs have been the most frequently used treatment for NLUTD in the clinical setting (Siddiqui et al., 2010; Madhuvrata et al., 2012). Although these drugs are effective in the improvement of cystometric capacity and bladder compliance (Madhuvrata et al., 2012), they are also associated with a high incidence of adverse drug reactions that often leads to treatment discontinuation (Nicholas et al., 2015). Therefore, it is essential that we identify alternative drugs that are both safe and effective.

Mirabegron is a β3-adrenergic agonist that is expected to become an efficient alternative to antimuscarinic agents due to its promising efficacy on the overactive bladder (Chapple et al., 2014). β-adrenoceptors can be divided into three subtypes: β1, β2, and β3 (Bylund et al., 1994). β3-receptors are predominately expressed in the heart, gastrointestinal tract, brain, prostate, and bladder detrusor (Ursino et al., 2009). However, because the β-adrenoceptors are widely expressed in the cardiovascular system, β-receptor agonists tend to induce adverse cardiovascular reactions. Because of its high selectivity toward β3-adrenoreceptors, mirabegron is rarely associated with complications (Korstanje et al., 2017). Previous studies have indicated that β3-adrenoceptor agonists exhibit the potential to cause human ureter relaxation (Matsumoto et al., 2013). The mechanisms of action by which mirabegron differs from anticholinergic drugs relate to the relaxation of the detrusor muscles in the storage phase; these effects occur *via* the stimulation of β3-adrenoreceptors. Previous studies have indicated that mirabegron was superior to antimuscarinic drugs in terms of cardiovascular complications (Rosa et al., 2018).

In the recent years, the role of mirabegron on NLUTD has attracted increasing levels of attention. For example, Beauval et al. proved that mirabegron treatment significantly improved micturition frequency and non-voiding contractions in a rat model of SCI (Beauval et al., 2015). In another study, Chen et al. reported the beneficial effects of mirabegron on the lower urinary tract symptoms of patients suffering from Parkinson’s disease and stroke (Chen and Kuo, 2019). Another retrospective chart review by Wöllner et al. concluded that mirabegron treatment has several advantages for patients with neurogenic detrusor overactivity (Wöllner and Pannek, 2016). Furthermore, Karakus et al. demonstrated that mirabegron is an effective and safe option for erectile function in men with an overactive bladder and erectile dysfunction (Karakus et al., 2021). Mullen et al. showed that mirabegron was effective in improving the urinary symptoms of patients with both overactive bladder and benign prostatic hyperplasia (Mullen and Kaplan, 2021).

We evaluated the treatment outcome of patients with several tools: the PPBC, I-QOL, and the Treatment Satisfaction-Visual Analog Scale (TS-VAS). PPBC is an evaluation form developed by the European Medical Evaluation Association to assess the global urinary incontinence problem and aims to report a subject’s subjective sensation of problems relating to the lower urinary tract (Coyne et al., 2006). The I-QoL, as a simple clinical investigation method, was originally designed to investigate the quality of life of women suffering from stress urinary incontinence (Patrick et al., 1999). The TS-VAS is used to record the subjective satisfaction of a patient with regard to their treatment. The results of these analyses were rated from 0 (none) to 100 (completely).

In our meta-analysis, we included four RCTs involving 245 patients who suffered from NLUTD. We assessed both the efficacy and safety of mirabegron for the treatment of NLUTD. The pooled results highlighted the significant superiority of mirabegron in terms of improving bladder compliance, urinary incontinence episodes, and I-QOL scores than placebo. In terms of PPBC and urinary urgency episodes, mirabegron therapy does not appear to differ from that of the placebo group. In a previous study, Krhut et al. found that mirabegron was superior to the placebo in terms of improving volume at the first detrusor contraction; it also improved TS-VAS and reduced urine leakage over 24 h (Krhut et al., 2018). In one previous RCT, the neurogenic bladder symptom score of a mirabegron group was significantly higher than the placebo group (Welk et al., 2018). With regard to safety, we found no significant difference between the mirabegron group and the placebo group in terms of the incidence of drug-related adverse events, arrhythmias, hypertension, and post-voiding residual volume. With this meta-analysis, we concluded that mirabegron can significantly improve the symptoms of NLUTD and has a superior clinical safety profile when compared with a placebo. These findings provide the basis for the continued use of mirabegron as an effective therapeutic strategy for the NLUTD.

Our meta-analysis has several strengths. First, the studies that we analyzed were all RCT; this means that the risk of bias was low. Second, to the best of our knowledge, very few previous reports have attempted to investigate the efficacy and safety of mirabegron for the treatment of NLUTD. Our study provides a strong support for the clinical use of mirabegron in NLUTD. However, there are also some limitations that need to be considered. First, the number of studies included in this analysis was inadequate and could have resulted in publication bias. To address this, our future research will focus on the most recent RCTs. Second, this study was not able to evaluate the long-term effects of mirabegron. As a result, our findings need to be confirmed by performing more high-quality RCTs.

## Conclusion

Our study indicated that mirabegron was effective in relieving NLUTD symptoms and exhibited a favorable safety profile.

## Data Availability

The original contributions presented in the study are included in the article/Supplementary Material; further inquiries can be directed to the corresponding authors.
